# Fold-change Response of Photosynthesis to Step Increases of Light Level

**DOI:** 10.1016/j.isci.2018.09.019

**Published:** 2018-09-26

**Authors:** Avichai Tendler, Bat Chen Wolf, Vivekanand Tiwari, Uri Alon, Avihai Danon

**Affiliations:** 1 Department of Molecular Cell Biology, Weizmann Institute of Science, Rehovot, Israel; 2 Department of Plant & Environmental Sciences, Weizmann Institute of Science, Rehovot, Israel

**Keywords:** Biological Sciences, Systems Biology, Plant Biology

## Abstract

Plants experience light intensity over several orders of magnitude. High light is stressful, and plants have several protective feedback mechanisms against this stress. Here we asked how plants respond to sudden rises at low ambient light, far below stressful levels. For this, we studied the fluorescence of excited chlorophyll *a* of photosystem II in *Arabidopsis thaliana* plants in response to step increases in light level at different background illuminations. We found a response at low-medium light with characteristics of a sensory system: fold-change detection (FCD), Weber law, and exact adaptation, in which the response depends only on relative, and not absolute, light changes. We tested various FCD circuits and provide evidence for an incoherent feedforward mechanism upstream of known stress response feedback loops. These findings suggest that plant photosynthesis may have a sensory modality for low light background that responds early to small light increases, to prepare for damaging high light levels.

## Highlights

• Chl *a* fluorescence responds to fold-change (FCD) in low-medium input light
• Identified fast feedforward (IFFL) regulation that depends on direct light input
• The direct sensing of input and FCD response are typical of sensory modules
• The IFFL precedes known feedback photoprotective regulation

## Graphical Abstract

**Figure:**
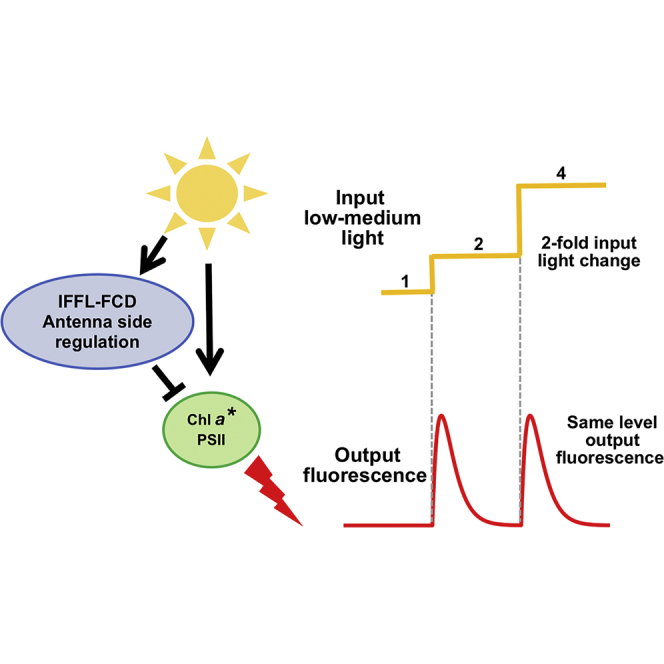

## Introduction

Photosynthesis converts sunlight into chemical energy that feeds the food chain. Photosynthesis must operate under several orders of magnitude of light input. The response of photosynthesis to light input is governed by at least two schemes. (1) Light is the source of energy for plants, and thus needs to be harvested efficiently. Sunlight energy is captured by the light-harvesting reactions and excites sequentially the photosystem (PS) II (PSII) and PSI chlorophyll (Chl)-containing reaction centers. These centers together generate electron transport that reduces NADP^+^ to form a transmembrane proton gradient that produces ATP. The NADPH and ATP produced by the light- reactions provide the chemical energy for the carbon fixation reactions. (2) Photosynthesis must protect itself from damaging effects of high light. High level of light results in excited Chl *a* side reactions that harm the PSII reaction center in a phenomenon termed photoinhibition (Jansen et al., 1999, Keren et al., 1997, Long et al., 1994). To circumvent photoinhibition, plants evolved a complex set of short- and long-term photoprotective mechanisms that protect the PSII reaction center by dissipating excessive light energy (Albanese et al., 2016, Allen et al., 1981, Demmig-Adams et al., 2012, Horton et al., 2008, Li et al., 2009, Minagawa, 2013, Muller et al., 2001, Niyogi and Truong, 2013, Pinnola and Bassi, 2018, Puthiyaveetil et al., 2017, Rochaix, 2014, Ruban, 2016, Schottler and Toth, 2014). The fast photoprotective mechanisms are under feedback-type regulation by the steep transmembrane proton gradient that is formed under excessive light levels (Armbruster et al., 2017, Li et al., 2009). The feedback regulation of the fast photoprotective mechanisms is thought to be mediated posttranslationally by structural or activity changes of existing regulatory proteins (Bassi and Caffarri, 2000, Brooks et al., 2014, Demmig-Adams, 1990, Horton et al., 1996).

The dynamic and unpredictable nature of input light and the environment in general adds to the complexity of the photosynthetic response (Chazdon et al., 1996, Pearcy, 1990). Plants evolved a plethora of short- and long-term mechanisms to respond properly to different magnitudes and dynamics of environmental changes. Recent studies showed that both short-term dynamic acclimation and long-term developmental acclimation were different in plants growing under fluctuating light conditions than in plants in constant light level (Alter et al., 2012, Athanasiou et al., 2010, Mullineaux et al., 2006, Okegawa et al., 2007, Retkute et al., 2015, Tikkanen et al., 2010, Vialet-Chabrand et al., 2017, Wagner et al., 2008, Walters, 2005, Yamori, 2016, Yin and Johnson, 2000). Special regulation was recently found also for photosynthetic responses to changes in the low and moderate light intensity range (Alter et al., 2012, Dangoor et al., 2012, Eliyahu et al., 2015, Finazzi et al., 2004, Golding et al., 2004, Naranjo et al., 2016, Nikkanen et al., 2016, Yamori et al., 2015).

A well-studied, nonintrusive measure of photosynthetic response to light changes is the PSII Chl *a* fluorescence, which reports on the level of excited Chl *a* (Baker, 2008, Krause and Weis, 1991, Stirbet et al., 2014). Chl *a* fluorescence level rises when the rate of photons harvested by the PSII antenna increases and excites the reaction center, and when more of the PSII primary acceptor Q_A_ becomes reduced, i.e., becomes “closed” to accepting electrons from the excited Chl *a*. Chl *a* fluorescence level decreases by photochemical quenching, i.e., when Q_A_ is oxidized by the subsequent electron transfer reactions, or by non-photochemical quenching via the action of the photoprotective mechanisms (Muller et al., 2001, Niyogi and Truong, 2013, Ruban, 2016). The photoprotective mechanisms regulate both key electron acceptors and the optical cross-sectional absorption of PSII (Foyer et al., 2012, Horton et al., 1996). The attenuation of photosynthetic electron transport is expected to increase fluorescence and that of the optical cross-sectional absorption of PSII is expected to decrease fluorescence.

In general, there are two main types of responses of biological systems to environmental changes: a response to absolute change and a response to relative change. Responses to absolute change in input signal are often found in stress response systems in which the response size matches the amplitude of the input signal. For example, a given amount of damage to proteins or DNA requires a proportional amount of repair enzymes. In contrast, a response to relative changes has thus far been found mainly in sensory systems. Response to relative changes results in increased sensitivity under lower backgrounds of input level, and can filter out noise in a relative manner to the background level (Goentoro et al., 2009, Shoval et al., 2010). Such systems usually show exact adaptation in which the output acclimates to the ambient signal (Alon et al., 1999, Barkai and Leibler, 1997).

A stringent type of response to relative changes is called fold-change detection (FCD), in which the entire response dynamics, including amplitude and response time, depend only on relative changes in input (Adler and Alon, 2018, Goentoro and Kirschner, 2009, Shoval et al., 2010). Accordingly, an input step of signal from level 1 to 2 yields exactly the same pulse of output response as a step from 2 to 4, because both steps have a 2-fold change. FCD has been found in systems including bacterial and amoeba chemotaxis (Lazova et al., 2011, Shoval et al., 2010), human vision (Shoval et al., 2010), *C*. *elegans* olfaction (Larsch et al., 2015) and mammalian signaling systems such as nuclear factor-κb (Lee et al., 2014) and transforming growth factor-β (Frick et al., 2017). FCD is such a stringent response that only a few types of specific circuits can provide FCD. These include the incoherent feedforward loop and specific nonlinear integral feedback loops (Adler et al., 2017, Alon, 2007, Goentoro et al., 2009, Shoval et al., 2010). These circuits can be differentiated based on input-output measurements (Adler et al., 2014, Rahi et al., 2017), providing a useful tool for understanding the type of mechanism at play (e.g. feedforward versus feedback), independently of a detailed elucidation of the underlying molecular mechanisms. When combined with nonintrusive assays, such as measuring the response of Chl *a* fluorescence to changes in input light, the input-output approach allows for analysis with little interference from the measuring devices. This is especially important for analyzing responses at the low light intensity range where even a small interference of the measuring device could influence the analysis.

Here, we studied the type of response of the photosynthetic system to input light step increases under low-moderate ambient light, far below stressful levels. For this purpose, we considered PSII Chl *a* fluorescence yield in *Arabidopsis thaliana*. We took an input-output approach to minimize the number of assumptions regarding the details of the underlying regulatory mechanisms. We presented the plants with steps of light with various magnitudes at different ambient light levels and measured the fluorescence signal. We found an approximate FCD response on the timescale of seconds at light levels below 160 $\text{μE}/{\text{m}}^{2}\text{s}$. FCD saturated at high light levels. We provide evidence that the circuit at play is an incoherent feedforward loop. These results show that the regulation of photosynthesis contains an additional mechanism with sensory-like features that together with known feedback loops photoprotects the plant from sudden increases in light level in the low-light regime.

## Results

### PSII Chl a Fluorescence Shows Fold-change Response in the Low to Medium Light Intensity Range

**Figure 1:**
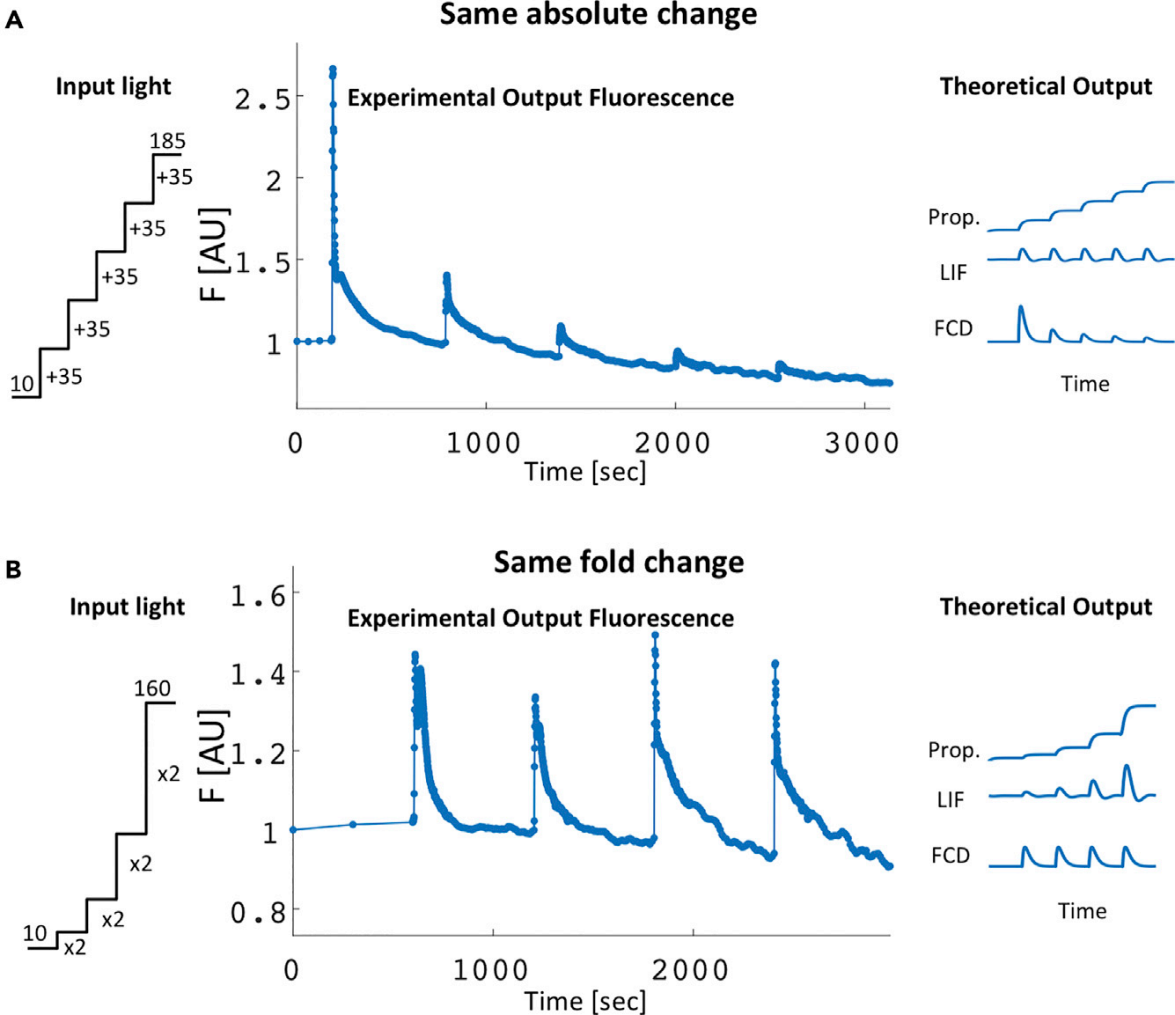
Chl *a* Fluorescence Shows Approximate Fold-Change Detection in Response to a Series of Input Light Steps (A and B) *A*. *thaliana* plants pre-adapted to 10 $\text{μE}/{\text{m}}^{2}\text{s}$ were presented with a series of 10-min spaced light steps (input light) with either (A) the same absolute change of 35 $\text{μE}/{\text{m}}^{2}\text{s}$ or (B) the same 2-fold change. The normalized fluorescence responses (experiment output fluorescence) are compared with three theoretical models (theoretical output): a proportional response in which output is proportional to the absolute change in input (Prop.), an adapting linear integral feedback loop (LIF) in which the output is proportional to the absolute change in input, and an incoherent feedforward loop fold-change detection model (FCD) in which the response is determined by the relative change in input. Results resembled the FCD model. Data represent the mean of 15 plants. Experimental standard errors were on the order of 3%.

To study the response of PSII Chl *a* fluorescence to small abrupt increases of light level, we presented *A*. *thaliana* plants with a series of uniform step increases of light level $\left(35\;\text{μE}/{\text{m}}^{2}\text{s}\right)$ that spanned the low to medium light intensity range $\left(10-185\;\text{μE}/{\text{m}}^{2}\text{s}\right)$ (Figure 1A, input light). The plants were kept for 10 min under the attained light level after each step to allow for short-term acclimation. We found that the response to each step of input light level was a pulse of fluorescence that reached its peak maximum within 5 s and then declined back to baseline within minutes (Figure 1A, experimental fluorescence output). Furthermore, the response to small light steps with the same absolute change $\left(35\;\text{μE}/{\text{m}}^{2}\text{s}\right)$ resulted in a series of fluorescence pulses with *decreasing* amplitudes. The largest response was seen for the first step, 10 to 45 $\text{μE}/{\text{m}}^{2}\text{s}$, and the smallest response for the last step, 150 to 185 $\text{μE}/{\text{m}}^{2}\text{s}$. A decreasing type of response is consistent more with regulation of optical cross-sectional absorption of PSII rather than regulation by availability of electron acceptors of PSII, which is expected to result in a similar response to similar absolute steps in light (linear integral feedback model [LIF] response). In addition, the fluorescence declined after each step back to the initial level before the first step increase of light level, a property called exact adaptation. The declining amplitudes of successive pulses and the exact adaptation indicated that photosynthesis is tightly regulated by light at the low-medium ambient light range.

To place this result in the context of theories, we simulated three well-studied models: a simple proportional response to input (Figure 1A, Prop.), a linear integral feedback model (LIF) that responds to absolute changes in input signal and has exact adaptation (Figure 1A, LIF), and an FCD model (Figure 1A, FCD). The FCD model, which shows declining pulses and exact adaptation for the absolute change steps, best matched the experimental observation (Figure 1A, experimental output fluorescence). These findings ruled out a response to absolute changes.

To test the possibility of FCD, we presented the plants with a series of steps with the same fold-change input. We used a series of step increases of light level with 2-fold change, resulting in absolute attained levels of 20, 40, 80, 160 $\text{μE}/{\text{m}}^{2}\text{s}$ (Figure 1B, input light). This series spanned a similar range of light intensities as before and had the same 10-min spacing between steps to allow for short-term acclimation. We found that the experimental response was a series of output fluorescence pulses with similar amplitude (Figure 1B, experimental output fluorescence), indicating that despite the fact that each step had a larger absolute change than the previous step, the output remained approximately the same.

The results of the fold-change input experiment contradicted the proportional and LIF models, and agreed with an FCD mechanism, including its exact adaptation property (Figure 1B, theoretical output), as did the results of the same-absolute-change experiment (Figure 1A). Together, the results suggest an approximate FCD property in the regulation of the response of photosynthesis to sudden rise in light level at the low-medium ambient light range. FCD and exact adaptation were found in many biological sensory systems (Alon et al., 1999, Bargmann, 2006, Barkai and Leibler, 1997, Eldar et al., 2002, Iglesias, 2012, Levchenko and Iglesias, 2002, Ma et al., 2009).

We noted that whereas the response amplitude matched the prediction of the FCD model, the shape of the output pulses in the fold-change input series was not exactly identical, and varied mainly during the phase of fluorescence decline (Figure 1B, experimental output fluorescence), suggesting that additional mechanisms might play a role in the decline of Chl *a* fluorescence (see below).

### The Chl a Fluorescence Response at Low to Medium Light Intensity Satisfies a Logarithmic Weber-Fechner Law

**Figure 2:**
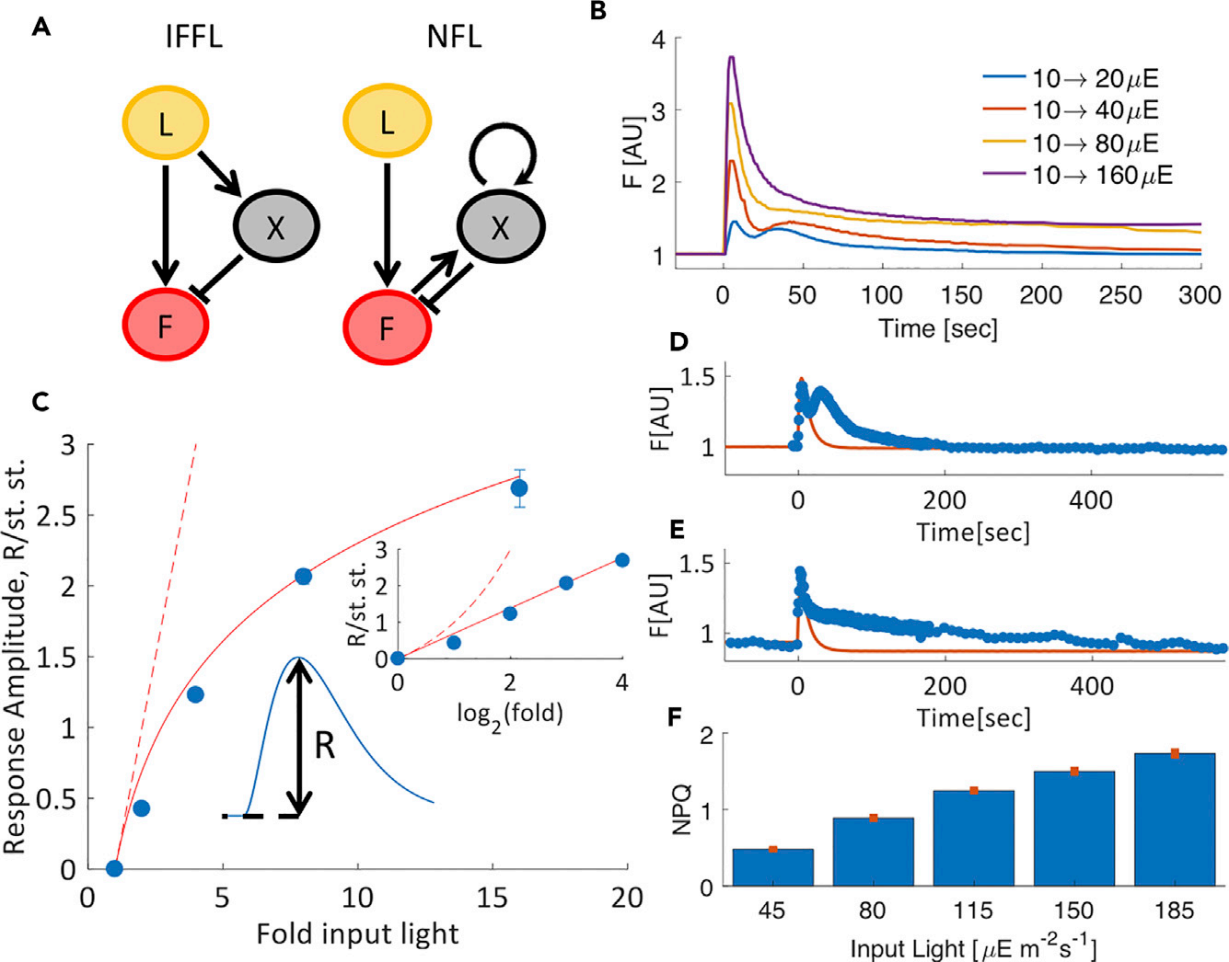
Chl *a* Fluorescence (F) Response Amplitude Depends Logarithmically on Fold-Change of Input Light (L) under Range of Low-Moderate Light Levels (A) The circuitry of the incoherent feedforward loop and nonlinear integral feedback loop theoretical models of FCD is presented. X is an internal variable; in the incoherent feedforward loop case, X is regulated directly by input light, whereas in the feedback loop case, X is regulated by the output fluorescence. (B) Normalized fluorescence responses to step increases of light from adaptation at 10 $\text{μE}/{\text{m}}^{2}\text{s}$ to differing levels from 20 to 160 $\text{μE}/{\text{m}}^{2}\text{s}$, generating fold-changes of 2, 4, 8, and 16. Each curve is the mean of 15 plants. (C) The fluorescence response amplitude depends logarithmically on the fold of the input light step, as in the theoretical incoherent feedforward loop model. Blue points are *A. thaliana* experimental data; error bars are standard errors over 15 plants, and are smaller than the marker size in some cases. Red curves are the predictions of the two theoretical models (solid line: ln(F) as predicted by incoherent feedforward loop; dashed line: F−1 as predicted by nonlinear integral feedback loop). Insert shows the same results in log scale. (D and E) Normalized fluorescence response amplitude and steady state matched the incoherent feedforward loop-FCD model (with carrying capacity that we introduce later), whereas the dynamics of the decline was not explained by the model and probably related to other regulatory processes. Blue points are experimental data (mean of 15 plants), and red curve is the theoretical model. The examples given are of steps (D) 10 to 20 $\text{μE}/{\text{m}}^{2}\text{s}$ and (E) 80 to 160 $\text{μE}/{\text{m}}^{2}\text{s}$. (F) Measurement of nonphotochemical quenching (NPQ) after exact adaptation was attained. Data points are the mean of 15 plants, error bars represent standard error of the mean.

There are two major types of FCD mechanisms: an incoherent feedforward loop and a nonlinear integral feedback loop (Figure 2A). The incoherent feedforward loop and feedback loop mechanisms are distinguished by their response amplitude dependence on fold-change (Adler et al., 2014, Shoval et al., 2010). The response amplitude is defined as the peak level of output minus the steady-state divided by the steady-state output. Nonlinear integral feedback loop circuits have linear or power-law dependence of response amplitude on input fold-change (also called Stevens law [Stevens, 1957]), whereas incoherent feedforward loop circuits predict logarithmic dependence called the Weber-Fechner law (Fechner, 1966, Ferrell, 2009, Weber et al., 1996).

To infer the type of circuit at play, we analyzed the relationship of the Chl *a* fluorescence response amplitude and the fold-change in input light level. For this purpose, we essayed input steps in which each set of plants was first acclimated for 30 min under 10 $\text{μE}/{\text{m}}^{2}\text{s}$ and then was treated with one step increase of light intensity to either 20, 40, 80, or 160 $\text{μE}/{\text{m}}^{2}\text{s}$, resulting in corresponding steps of 2-, 4-, 8-, and 16-fold increased light intensity. As expected from both the FCD models, the response amplitude increased with the fold-change of the input steps (Figure 2B). We extracted the dependence of response amplitude on the fold-change in input and found a logarithmic dependence (Figure 2C), suggesting an incoherent feedforward loop rather than a feedback loop mechanism. To further test the possibility of incoherent feedforward loop we used an independent experimental test developed by Rahi et al. (Rahi et al., 2017). In this test, that expected differences in the state of an internal variable of the two mechanisms could result in different response amplitudes to two consecutive pulses of inputs. We found that the response amplitude to the second pulse matched the theoretical response of incoherent feedforward loop rather than that of a feedback loop mechanism (Supplemental Information, two-pulse experiment also suggests a feedforward circuit, Figures S7 and S8). Collectively, these findings suggest an incoherent feedforward loop-type FCD mechanism at low-moderate light intensities.

Each pulse decline phase contained a secondary shoulder of different magnitude (Figure 2B). Fitting of the incoherent feedforward loop-FCD model described well the initial fluorescence rise, the amplitude peak, and the adapted levels of the response, but did not match the dynamics of return to baseline after the peak (Figures 2D and 2E). At low light levels a second peak was found, and at high light levels a more gradual return was detected. This suggested the participation of additional fluorescence quenching mechanisms in the process of return to the initial fluorescence level (Figure S10). The prime candidates for such mechanisms are the fast feedback-type regulations that have been characterized in this system (Albanese et al., 2016, Allen et al., 1981, Demmig-Adams et al., 2012, Horton et al., 2008, Li et al., 2009, Minagawa, 2013, Muller et al., 2001, Niyogi and Truong, 2013, Puthiyaveetil et al., 2017, Rochaix, 2014, Ruban, 2016, Schottler and Toth, 2014).

To assay the contribution of the non-photochemical fluorescence quenching mechanisms to the reestablishment of the initial fluorescence level after each light step, we measured the maximum fluorescence yield before the start of the experiment and 10 min after each light step increase $\left(35\;\text{μE}/{\text{m}}^{2}\text{s}\right)$. We found increasing level of non-photochemical fluorescence quenching in plants after their return to the initial fluorescence level (Figure 2F). This finding corroborated the contribution of the fast non-photochemical fluorescence quenching mechanisms to the regulation of the fluorescence response during the phase of reestablishment of the initial level.

### The Chl a Fluorescence Fold-change Response Is Saturated under High Light Levels

**Figure 3:**
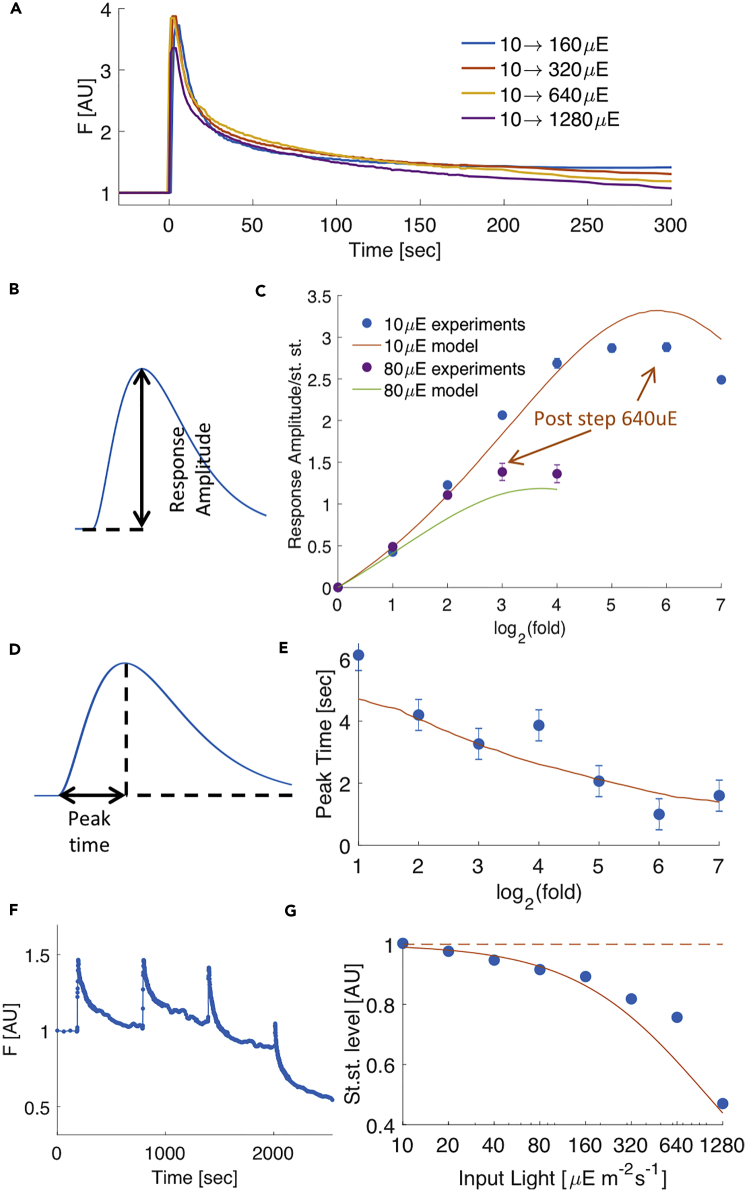
FCD Saturated under High Input Light; This Can Be Explained Using Incoherent Feedforward Loop with Carrying Capacity of Light (A) Normalized fluorescence responses to steps from an adapted light level of 10 $\text{μE}/{\text{m}}^{2}\text{s}$ to various levels from 160 to 1,280 $\text{μE}/{\text{m}}^{2}\text{s}$. Each curve is the mean of 15 plants. (B) Illustration of response amplitude. (C) Response amplitude as a function of fold-change in light for steps from an adapted level of 10 $\text{μE}/{\text{m}}^{2}\text{s}$ (blue dots) and for steps from an adapted level of 80 $\text{μE}/{\text{m}}^{2}\text{s}$ (purple dots). Full lines: incoherent feedforward loop model with saturation of Chl *a* fluorescence. Data represent the mean of 15 plants in three experiments, and error bars are standard error of the mean over experiments. (D) Illustration of peak time. (E) Peak time as a function of fold-change in light (dots) is a decreasing function, well-described by the saturated incoherent feedforward loop model (full line). Data represent the mean of 15 plants in three experiments; error bars follow from the temporal measurement resolution. (F) Response to series of light steps from 80 to 160 to 320 to 640 to 1,280 $\text{μE}/{\text{m}}^{2}\text{s}$ showing decreasing steady-state levels. (G) Steady-state fluorescence decreases at high light levels. Full line: saturated incoherent feedforward loop model, dashed line: saturated nonlinear integral feedback loop model.

The fluorescence response amplitude of plants treated with fold-change step increases of light level in the low to moderate light intensity range matched the output of an incoherent feedforward loop-type FCD model, even when the largest 16-fold step increase $\left(10-160\text{μE}/{\text{m}}^{2}\text{s}\right)$ was included in the analysis. It was therefore interesting to study whether plants show the same type of response to fold-change step increases of the higher range of light intensities, above 160 $\text{μE}/{\text{m}}^{2}\text{s}$. For this purpose, we essayed input steps in which each set of plants was first acclimated for 30 min to 10 $\text{μE}/{\text{m}}^{2}\text{s}$, and, then, was treated with a step increase of light intensity to 160, 320, 640, or 1,280 $\text{μE}/{\text{m}}^{2}\text{s}$, resulting in corresponding steps of 16-, 32-, 64-, and 128-fold increased light intensity (Figure 3A). We found that the logarithmic rise of response amplitude (Figure 3B) saturated above 160 $\text{μE}/{\text{m}}^{2}\text{s}$, and even dropped slightly at higher light levels (Figure 3C, blue dots).

We next asked whether the saturation of the logarithmic rise was a result of higher ambient light level, above 160 $\text{μE}/{\text{m}}^{2}\text{s}$, or a result of the larger input steps, above 16-fold step increase. For this purpose, we studied Chl *a* fluorescence response of plants that were acclimated first to 80 $\text{μE}/{\text{m}}^{2}\text{s}$ light intensity to increasing fold-change steps of light level (Figure 3C, purple dots). A comparison of the response of plants acclimated to either 10 or 80 $\text{μE}/{\text{m}}^{2}\text{s}$ light levels revealed that saturation was achieved at different levels of fold-change in input. Plants acclimated to 10 $\text{μE}/{\text{m}}^{2}\text{s}$ reached maximal fluorescence amplitude above the 32-fold step (320 $\text{μE}/{\text{m}}^{2}\text{s}$ absolute light level), whereas those acclimated to 80 $\text{μE}/{\text{m}}^{2}\text{s}$ reached the maximal response amplitude already at the 8-fold step (640 $\text{μE}/{\text{m}}^{2}\text{s}$ absolute light level). This suggests that the *saturation* of the FCD is dependent on the post-step ambient light intensity of 320–640 $\text{μE}/{\text{m}}^{2}\text{s}$ rather than the fold-increase of light.

To model the saturation effect, we included in the incoherent feedforward loop-type FCD model a Michaelis-Menten carrying capacity of Chl *a* fluorescence yield by changing the input from L to L/(K + L), where K is the midpoint saturation constant. We used a midpoint of K = 1,000 $\text{μE}/{\text{m}}^{2}\text{s}$. We found that the modified incoherent feedforward loop-type FCD model explained the response amplitude under the range of low to medium light intensity, its saturation under high light levels (solid lines Figure 3C), as well as the time it takes to reach the peak amplitude (peak time) of the various steps (Figures 3D and 3E) under both low and high light levels. We note that the mild reduction in peak time with increased fold-change (Figure 3E) in input is a general feature of FCD models (Adler et al., 2014).

Next, we tested whether the exact adaptation that was observed in the fluorescence response under the ambient low to moderate light intensity range is maintained also at high light levels. For this purpose, we presented the plants with a series of input steps rising 2-fold from 80 to 1,280 $\text{μE}/{\text{m}}^{2}\text{s}$. We found that when post-step ambient light levels exceeded 160 $\text{μE}/{\text{m}}^{2}\text{s}$, the fluorescence level attained at the end of each 10-min step was lower than the pre-step level (Figures 3F and 3G), indicating that the exact adaptation property of the FCD was lost at high light levels. We note that the revised saturated incoherent feedforward loop-type FCD model with the above parameters predicted this loss of exact adaptation (solid line in Figure 3G). Furthermore, feedback models for FCD, such as nonlinear integral feedback loop (NFL), do not show such a change in the exact adaptation property even when saturation of the input is added (dashed line in Figure 3G and Supplemental Information, exact adaptation is not abolished for NFL). This adds additional support for the incoherent feedforward loop mechanism for FCD.

**Figure 4:**
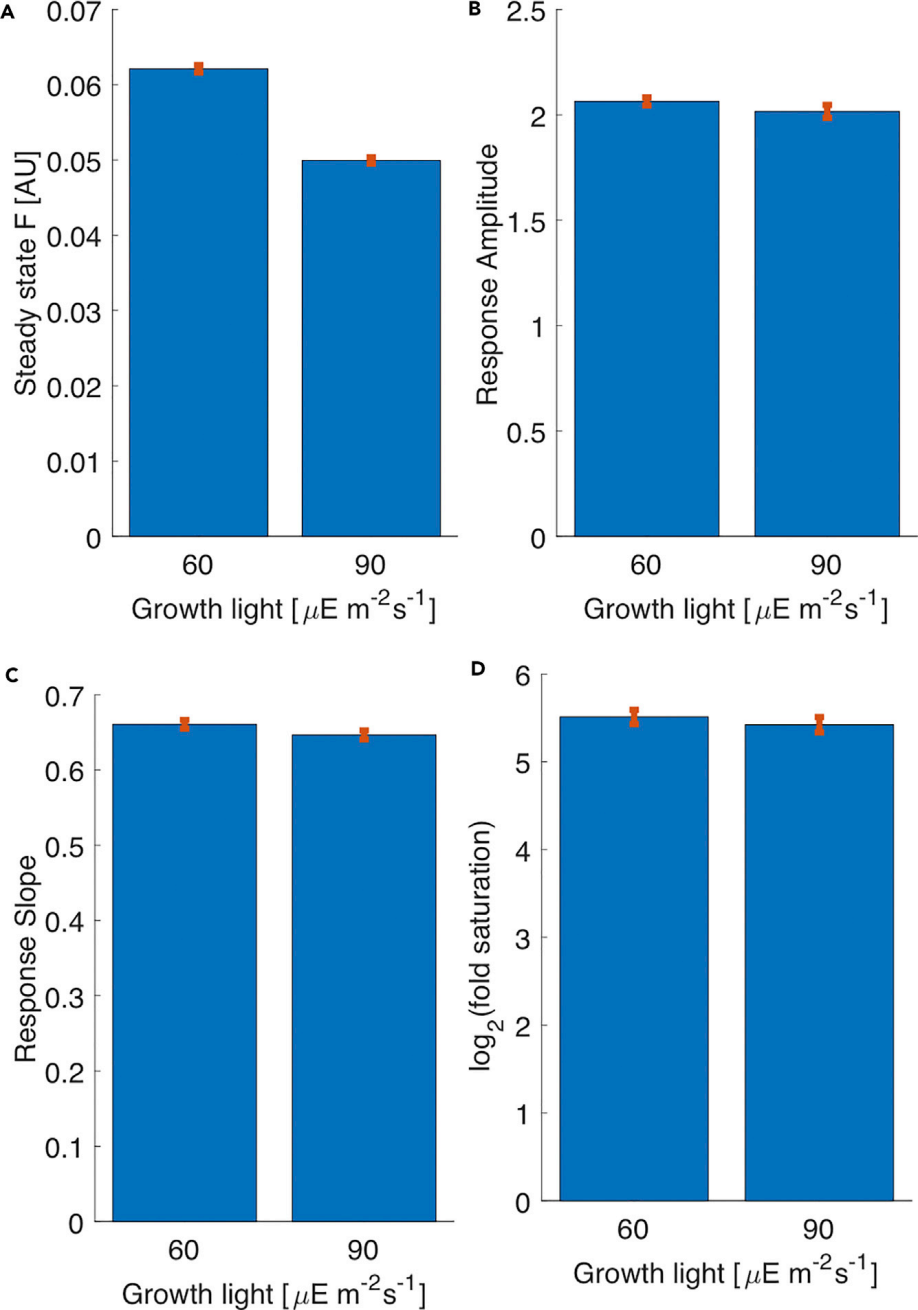
FCD Properties Are Robust to Changes in Growth Light (A) Steady-state fluorescence level changes significantly (25%) between growth light of 60 and 90 $\text{μE}/{\text{m}}^{2}\text{s}$. (B) Response amplitude changed marginally (2%) between growth lights. Response amplitude were computed based on the 8-fold experiment. (C) Slope of the response amplitude dependence on log-fold changed marginally (2%) between growth lights. (D) Fold under which FCD saturated changed marginally (2%) between growth lights. Subplots C-D in this figure were computed based on 15 plants from 3 repeats in the 7 different fold change experiments, as presented in Fig3C. In all subplots bars represent means and error bars are standard error of the mean.

Finally, we tested whether the properties of the incoherent feedforward loop-type FCD model are maintained in plants that were grown in 50% higher light intensity $\left(90\;\text{μE}/{\text{m}}^{2}\text{s}\right)$. We found that in spite of different fluorescence values at steady state in plants grown under 60 or 90 $\text{μE}/{\text{m}}^{2}\text{s}$, all tested FCD properties, the response amplitude, the slope of the response amplitude dependence on log-fold, and the fold under which the FCD saturated, were similar in plants under both experimental conditions (Figures 4 and S9).

## Discussion

We studied the response of PSII Chl *a* fluorescence in *A*. *thaliana* plants to light steps that go between different initial and final light levels. This allowed us to ask whether the response is to absolute or relative changes. We found that at low-moderate light levels, the fluorescence response was approximately FCD, with a pulse amplitude and response time that depended on relative changes in light rather than absolute changes, and a return to the initial fluorescence level. We identified the FCD mechanism as incoherent feedforward loop based on the logarithmic dependence of the amplitude on the fold-change in light and on the Rahi test that is based on differences of the response amplitudes of the incoherent feedforward loop and the nonlinear negative feedback loop to two consecutive pulses of input (Rahi et al., 2017). At higher light levels, the response saturated and showed loss of exact adaptation, supporting an incoherent feedforward loop mechanism with a carrying capacity that saturates at high light levels. This points to a sensory-like modality for low-medium light levels in plant photosynthesis.

This study suggests feedforward circuitry in addition to the feedback photoprotective mechanisms identified thus far. One may ask what might be the benefit of feedforward regulation, added to the feedback regulation. A feedforward circuit depends directly on the input, and thus has the advantage of responding quickly, even before a measurable outcome of the change appears. However, because a feedforward circuit depends entirely on the input, it lacks the ability to adjust itself when downstream factors are perturbed. In contrast, a feedback circuit responds later in time, only after a measurable outcome has appeared, but can adjust the regulated response more accurately based on the developing outcome. Thus, we hypothesize that the feedforward loop of the FCD is important to an immediate anticipatory response of photosynthesis to abrupt light increases, avoiding delays that could put the plant at risk. The multiple feedback mechanisms that are regulated by a threshold level of thylakoid lumen proton concentration, by redox changes of the photosynthetic electron transfer chain, or by stromal signals (Armbruster et al., 2017, Dangoor et al., 2012, Golding et al., 2004, Li et al., 2009, Naranjo et al., 2016, Pinnola and Bassi, 2018) are important to adjust the regulated response with high accuracy based on multiple parameters that report on the developing photosynthetic outcome.

Many naturally occurring light fluctuations faced by plants, such as canopy shifting and mixing and cloud cover, are proportional to background light (Pearcy, 1990). For example, a shadow of a leaf or a cloud typically reduces a certain fraction of the ambient light level. An important feature of the FCD circuit is that it can accurately detect proportional changes in the low-light regime. A 10 $\text{μE}/{\text{m}}^{2}\text{s}$ change in light can be significant and informative to plants acclimated to background of 10 or 20 $\text{μE}/{\text{m}}^{2}\text{s}$, whereas the same 10 $\text{μE}/{\text{m}}^{2}\text{s}$ change might be simply noise in the environment to plants acclimated to a background of 160 $\text{μE}/{\text{m}}^{2}\text{s}$. In addition to its fast response, the incoherent feedforward loop-FCD mechanism found here could allow the plant to respond appropriately to both signals: a strong response at low light background and a weak one under high background. This could be important for plants acclimated to low light conditions, which are known to have increased light harvesting capacity, and thus are more vulnerable to sudden increases of light level (Demmig-Adams and Adams, 1992, Walters, 2005). The findings of this study agree with the recent hypothesis that the fast non-photochemical quenching mechanisms evolved to ensure a prompt and substantial response of the photosynthetic light harvesting to sudden exposure to high light (Ruban, 2015).

In our proposed working model of an incoherent feedforward loop upstream of feedback circuits (Figure S10), the fluorescence response is coordinated early by an incoherent feedforward loop-FCD circuitry that delineates the pulse-shape response with an amplitude that depends on the fold-change of input light (Figures 2B and 2C). The dependence of incoherent feedforward loop on direct light signal and its influence on the earliest response phase suggest a mechanism associated with light harvesting and regulating the optical cross-sectional absorption of PSII. Downstream of this incoherent feedforward loop-FCD circuit are the feedback-type circuitry that control the timing of the fluorescence decline (Figures 2D and 2E). Thus, when the incoherent feedforward loop-FCD regulation senses a sudden input light change, it initiates a fast but imprecise predictive heuristic of the fold-change response, such that a small change in actinic light triggers large response in plants at low ambient light and small response in plants at high ambient light. Subsequently, the feedback circuitry is activated and acts to adjust the response based on changes in threshold level of thylakoid lumen proton concentration, redox changes of the photosynthetic electron transfer chain, or stromal signals. The regulation by the incoherent feedforward loop-FCD and feedback circuitry maintains the exact adaptation feature of FCD as indicated by the return of the fluorescence level to its initial value after each step increase of input light (see Supplemental Information: A Feedback Mechanism Can Work in Concert with the FCD-IFFL circuit). We hypothesize that the biological purpose of the exact adaptation of Chl *a* fluorescence is to maintain proper dynamic balance between the rates of light harvesting and photochemistry that minimizes the deleterious side reactions of excited Chl *a* in spite of sudden increases of light intensity.

Future work is needed to decipher the molecular mechanism for FCD in photosynthesis. It would be interesting to see if an incoherent feedforward loop is indeed at play, and to discover the internal node that carries the memory of the background light. More generally, this study suggests that a similar physiological input-output approach could be used to discover FCD in other biological systems, and to differentiate between FCD mechanisms, using response amplitude tests. Just as photosynthesis is shown here to harbor a sensory-like ability at low light levels, it will be fascinating to see if there are additional low-signal sensory-like abilities in plants.

### Limitations of Study

We took an input-output approach to understand Chl *a* florescence in wild-type *Arabidopsis thaliana* plants. This study does not include known mutants of feedback regulation and, thus, can only infer the circuit at play, and is yet to reveal a specific molecular mechanism responsible for this incoherent feedforward loop-FCD circuit.

## Methods

All methods can be found in the accompanying Transparent Methods supplemental file.

## Supplemental Information

Document S1. Transparent Methods and Figures S1–S10
