# Supplementary material for: Fold-change Response of Photosynthesis to Step Increases of Light Level
Source: iScience. 2018 Sep 26;8:126–37. doi: 10.1016/j.isci.2018.09.019 (PMC6176854; doi:10.1016/j.isci.2018.09.019)
Supplement: Document S1. Transparent Methods and Figures S1–S10 [file mmc1.pdf]

ISCI, Volume *8*

Supplemental Information

Fold-change Response of Photosynthesis to Step Increases of Light Level

Avichai Tendler, Bat Chen Wolf, Vivekanand Tiwari, Uri Alon, and Avihai Danon

Transparent Methods

Plant material and growth conditions

Arabidopsis thaliana var. Columbia (Col-0) was grown in ambient air on solid half-strength Murashige and Skoog medium in 0.8% agar plates. Plants were grown under a 8/16 h light/dark cycle at 20°C/18°C, respectively, at a light intensity of 60 $\mu E/m^2 s$ for 3-4 weeks.

Chlorophyll fluorescence

Chl *a* Fluorescence was measured in three to four-week old plants (n=15 per measurement) after the 16-h of dark period, using an Imaging pulse amplitude modulation (PAM) chlorophyll fluorometer (Heinz Walz GmbH). Plants were first equilibrated for 30 min to 10 or 80 $\frac{\mu\mathrm{E}}{\mathrm{m}^2\mathrm{s}}$, as indicated in text, in the Imaging PAM chlorophyll fluorometer and then treated with sequential 10 min periods of different levels of light intensity, as indicated in text. Three biological repeats were obtained for each experiment.

Chlorophyll fluorescence quenching

Plants were pre-equilibrated to 10 $\frac{\mu\mathrm{E}}{\mathrm{m}^2\mathrm{s}}$ light for 30 min and then input light was increased by 35 $\frac{\mu\mathrm{E}}{\mathrm{m}^2\mathrm{s}}$ at successive 10 min steps. The Fm was recorded at the end of each 10 min. step. NPQ was calculated by the formula (NPQ= Fm-Fm'/Fm').

Theoretical models

To model a proportional response system in Fig. 1, we used $\frac{dF}{dt} = L - F$, where L is the input light and F is the output fluorescence. For adapting absolute response we used linear integral feedback $\frac{dX}{dt} = F - 1; \frac{dF}{dt} = L - X - F$, here X is an internal variable. For FCD we used type 1 incoherent feedforward loop $\frac{dX}{dt} = L - X; \frac{dF}{dt} = \frac{L}{X} - F$. A nonlinear integral feedback loop circuit was modeled using the equations $\frac{dX}{dt} = X(F-1); \frac{dF}{dt} = \frac{L}{X} - F$ (Shoval et al., 2010; Somvanshi et al., 2015; Yi et al., 2000).

Fitting

To include in the model the saturation of FCD at high light levels, we used a saturated incoherent feedforward loop circuit: $\frac{dX}{dt} = L - \frac{X}{T_s}; \frac{dF}{dt} = \frac{L}{(L+k)X} - \frac{F}{T_s}$. Here k is the halfway saturation point, T_s represent scaling of the time axis. Its steady state level is $F_{st} = \frac{1}{L+k}$, this is almost independent of L for low light

levels L<<K. All fits in this paper (Fig. 2D,E, Two fits of Fig. 3C, Fig. 3E,G) used this model with the same parameters, chosen by visual inspection to be $k = 1000[\frac{\mu E}{m^2 sec}]$ and $T_s = 3.5[sec]$. The corresponding saturated nonlinear integral feedback loop circuit is $\frac{dX}{dt} = X(F - \frac{1}{T_s}); \frac{dF}{dt} = \frac{L}{(L+k)X} - F$. It does not show breakdown of exact adaptation because its steady state is always $F_{st} = \frac{1}{T_s}$. See below for a more detailed description of this circuit.

Data analysis

Raw fluorescence traces included rare spurious spikes (raw data is shown in Fig. S1). We removed these spikes by smoothing the traces by a median filter with window size of 5. Pulse amplitude and peak times were calculated on these smoothed signals (using data without smoothing showed similar results, as did using filter window sizes ranging from 1 to 10, Fig. S2A). Response amplitude was defined as the $\frac{max\,(F) - Fst.st}{Fst.st}$, steady state was defined as the mean of the 5 time points before the step. Peak times were computed as the time after the step at which fluorescence reaches its maximal value. In the case of two peaks as happens in at low light, we used the first peak to determine response time.

Exact adaptation is not abolished for nonlinear integral feedback loop

The nonlinear integral feedback loop with carrying capacity with all standard parameters is defined by the equations:

$$\frac{dX}{dt} = \frac{\epsilon X}{F_s}(F - F_s)$$

$$\frac{dF}{dt} = \frac{L}{(L+k)X} - \frac{F}{T_s}$$

In addition to the carrying capacity k and the timescale parameter T_s, there is also an output scale parameter F_s and a dimensionless parameter ϵ responsible for the impact of the internal variable. Note that the steady state of this more general circuit is still independent of input light:

$$F_{st} = F_s$$

This contradicts what was found in our photosynthetic experiments.

Incoherent feedforward loop with all natural parameters

We used in the main text an incoherent feedforward loop with two parameters T_S which is responsible for the timescale of the circuit and k which is its carrying capacity. For completeness we mention here that the general circuit will consists of two more parameters, which we did not manipulate in this paper. F_s takes into account the scale of the output fluorescence and ϵ which is a dimensionless parameter. The general equations are hence:

$$\frac{dX}{dt} = \frac{\epsilon}{F_s}(L - \frac{F_s X}{T_s})$$

$$\frac{dF}{dt} = \frac{L}{(L+k)X} - \frac{F}{T_s}$$

We explain here the meaning of the parameters. A scaling of the time parameter $t \to \hat{t} = \alpha t$, results in the following changes in the equations: $\hat{X} = \alpha X$ and $\widehat{T_S} = \alpha T_S$. The other parameters do not scale with time. Hence, T_s is the parameter responsible for time scaling of the input and output. There is also the transformation of the internal variable, but it does not change the dynamics of the input and output.

Similarly, scaling F $\hat{F} = \beta F$ result in the following scaling of the equations: $\hat{X} = \frac{X}{\beta}$ and $F_s = \beta F_s$. The other parameters do not scale with fluorescence. Hence F_s represents the scale of fluorescence and accounts for the vertical scale of the output. Note that since florescence is an extensive quantity defined up to scale in our experiments, there is no need to fit the parameter F_S.

The parameter k is the carrying capacity. Note that for FCD circuit in general there is an independence of scale of input, therefore there is no such input scale parameter for these circuits. In our case, there is a carrying capacity of the input, therefore introducing an extra parameter to the circuit.

The parameter ϵ is a single dimensionless parameter characterizing the exact dynamics of the feedforward loop circuit. We find that $\epsilon = 1$ provides a good description. See also (Adler et al., 2017).

Raw data contains spurious spikes

In the main text Figs. 1, 2A,D,E and 3A,D show raw data smoothed with moving median filter of length 5. The reason this filter was applied is that raw data contains spurious spikes. Fig. S1A-C shows three examples of mean raw data of experiments without smoothing, revealing different spike severities. The spikes are not artifacts of a specific plant, rather they appear for all plants in a given experiment at the same time-points (Fig. S1D), suggesting a measurement artifact of the experimental system.

Results are independent of smoothing filter length

Some of the results in the main text were computed on the data filtered using moving median of length 5. We tested moving median of length 1 (no smoothing) to 10 (more pronounced smoothing). Since the smoothing decreases the maximum, the stronger the smoothing the weaker the response amplitude is. The smoothing does not change qualitatively the results, although there is a small quantitative change (Fig. S2A). The parameters of the model in the main text were tuned (manually) according to the curves smoothed of length 5.

To check the dependency of parameters in the level of smoothing, we also examined the model prediction under different values of model parameters T_s (Fig. S2B) and k (Fig. S2C). In general, increasing the smoothing parameter will result in slightly lower T_s and k. These plots also give some grasp on the expressivity of the model under different parameters. Artifacts in these plots follows from numerical errors in simulating the differential equations.

The main source of variation between plants in the experiments is a global scale factor

Fluorescence is defined up to a global scale factors, and plants dynamics differ from one another mostly by a global scale factor, an example of this phenomenon is seen in Fig. S1D, which shows the dynamics of individual plants in one of our experiments, the plants dynamics are proportional to one another.

To further illustrate the existence of a global scale factor we took the time series of one experiment and correlate the 15 individual plants. All pairwise correlations were above 0.8, showing that the dynamics closely resembles each other up to this global scaling (Fig. S3). This result is typical to the experiments performed in this work.

As a corollary of this property, we obtain that relative quantities are much more robust in our experiments. Relative quantities are ones which depends on ratio of quantities which measure fluorescence. For example, consider an experiment of step from $80\frac{\mu E}{m^2 sec}$ to $160\frac{\mu E}{m^2 sec}$. The steady states after adaptation to $80\frac{\mu E}{m^2 sec}$ vary by 14% between the 15 individual plants, the responses after the step also vary by 14%, but the ratio of response over steady state vary by only 3%. Response amplitudes are therefore good quantities to extract from such experiments, since they depend only on a ratio of quantities.

Individual plants behave similarly in all experiments

The data presented in the main text is a mean of 15 plants from an experiment. We show here that the patterns found while taking the mean are representative of the individuals plant. All plants behave similarly, and follow a similar pattern as the mean. Also, results of the three repeats of an experiment are similar to one another. Fig. S4, Fig. S5 and Fig. S6 show it for the experiments of constant absolute change, constant fold change and different steps, respectively.

Two pulse experiment also suggests a feedforward circuit

To obtain further evidence for the mechanism, we used a recently suggested method to distinguish incoherent feedforward loop (IFFL) and nonlinear negative feedback loop (NFL) based on input-output only (Rahi et al., 2017). It was discovered that the two mechanisms can be distinguished, at least under some range of parameters, based on the response amplitude to two consecutive pulses of inputs. Whereas the amplitude might be similar in the response to the first pulse, a difference is expected in the response to the second pulse. This is because of the different states of the internal variable which is not measured directly.

Let T denote the period of the pulse input (distance between consecutive pulses), and d the duration of one pulse, two main properties were found which distinguish between NFL and IFFL (Fig. S7):

1. NFL can show period skipping, i.e. it might not react to the second step, this might occur under some range of T,d and internal circuit parameters.
2. When increasing d, IFFL shows decreased response strength, this is not true for NFL.

Taking these features into account, we designed another experiment to infer the circuitry of the system. Dark adapted plants were exposed to $20\,\frac{\mu E}{m^2 s}$ light for 10min, then to a first pulse of $55\,\frac{\mu E}{m^2 s}$ for duration d, back to $20\,\frac{\mu E}{m^2 s}$ for T-d and again a second pulse for duration d. This repeated itself 12 times for different values of T and d. Between each repeat there was 10 min of $20\,\frac{\mu E}{m^2 s}$ exposure to let the system adapts back to its steady state.

The input and the mean output of 6 WT plants are shown in Fig. 4C. We can see that the second output peak is always smaller than the first peak, we can also see that there is no period skipping, since there is always a second peak. These findings further support the IFFL loop for the pulse amplitude.

We also found that for constant T, the second peak decreases as we increase d, also a prediction of IFFL circuitry (Fig. 4D).

We conclude from both the logarithmic Weber law, the absence of period skipping and the decreasing of second peak for increased d, that the control of fluorescence is IFFL.

A feedback mechanism can work in concert with the FCD-IFFL circuit

In this work we found evidence for an FCD-IFFL regulation involved in the fluorescence regulation. There are also known feedback mechanisms for photosynthetic regulation already characterized. Here we give theoretical examples of how we can combine a feedback and a feedforward mechanism keeping the FCD property intact.

One way to approach this question is to follow the principle of (Shoval et al., 2010), the intermediate variables can scale with the input, while for the output variable the scaling should cancel, let L denote the input, X, Y are intermediate variables and F is the output. Let:

$$\dot{X} = f(X, Y, F, L)$$

$$\dot{Y} = g(X, Y, F, L)$$

$$\dot{F} = h(X, Y, F, L)$$

Assume we have exact adaptation to the steady state output $F = F_0$ and that:

$$f(pX, pY, F, pL) = pf(X, Y, F, L)$$

$$g(pX, pY, F, pL) = pg(X, Y, F, L)$$

$$h(pX, pY, F, pL) = ph(X, Y, F, L)$$

Then we get FCD. For example, we can construct the following FCD circuit:

$$\dot{X} = L - X$$

$$\dot{Y} = Y(F - 1)$$

$$\dot{F} = \frac{L}{X^{0.5}Y^{0.5}} - F$$

This system is invariant under the transformation $L \to pL, X \to pX, Y \to pY, F \to F$. X here in an intermediate variable for feedforward regulation and Y is an intermediate variable for feedback regulation.

Interestingly, the experimental dynamics typically consists of fast response, fast initial partial adaptation and slow final adaptation, as in the experimental results in Fig. 2E. By adding a slow timescale to the feedback variable Y we can recover this form of dynamics (Fig. S10).

Supplementary figures

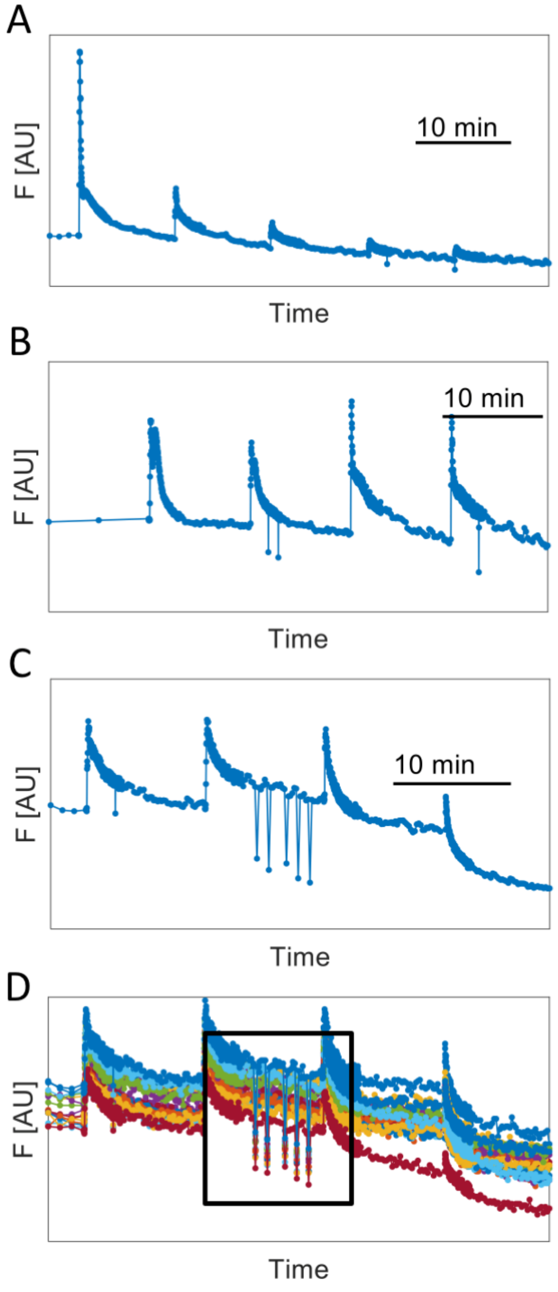

Figure S1. Raw data contains spurious spikes (Related to Figure 1). Raw data from three experiments is shown here as examples of the spikes phenomenon. (A) Input light was changed in constant absolute steps. (B) Input light was changed in constant fold steps under low light. (C) Input light was changed in constant fold steps under high light. (D) The individual plants in the experiment of panel C. The spikes exist for all plants at the same time points, probably due to an error in measurement.

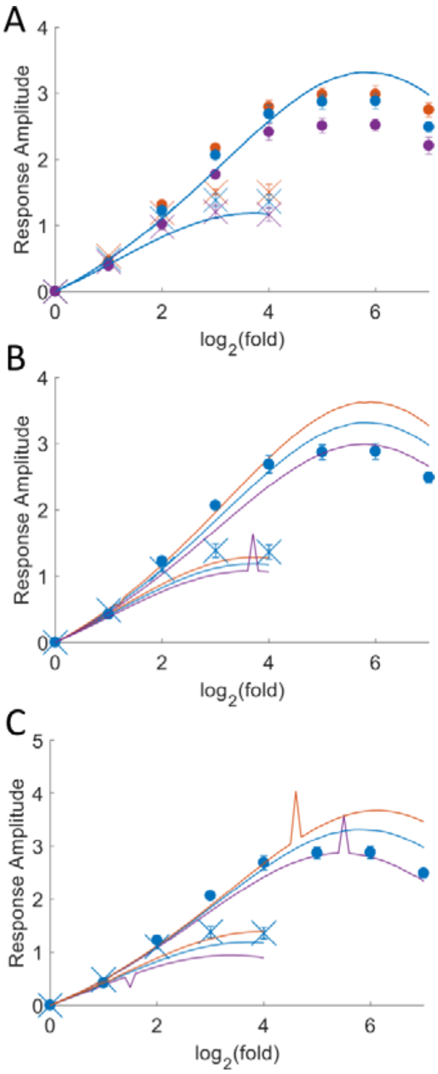

Figure S2. The smoothing does not change the results significantly (Related to figure 3C). (A) Response amplitude dependency on fold for different moving median smoothing kernels. Orange – no smoothing, blue – kernel of length 5 used in the main text, purple – kernel of length 10. Dots shows data for experiments of steps from 10uE, x are data for experiments from 80uE. There is a small quantitative difference between smoothing kernels, but qualitative behavior is the same. (B) The variation in model prediction as function of the parameter T_S. Plots show $T_s = 4[sec]$ (orange), $T_s = 3.5[sec]$ (blue, as used in the main text) and $T_s = 3\ [sec]$ (purple). (C) The variation in model prediction as function of the carrying capacity k. Plots show $k = 1300[\frac{\mu E}{m^2 sec}]$ (orange), $k = 1000\ \left[\frac{\mu E}{m^2 sec}\right]$(blue, as used in the main text) and $k = 700[\frac{\mu E}{m^2 sec}]$ (purple).

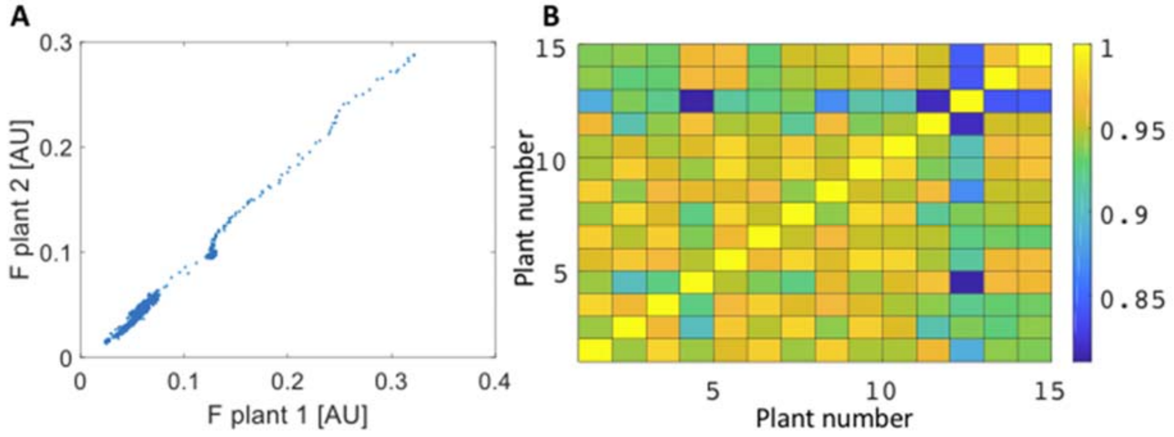

Figure S3. There is a global scale factor accounted for the main variation in our experiments (Related to figure 1). A) The plot shows two plants from the same experiments, the fluorescence values across time points are highly correlated. B) Correlation coefficients for all pairs of plants (from 1 to 15) from the experiment, all correlations are above 0.8.

Fig S4

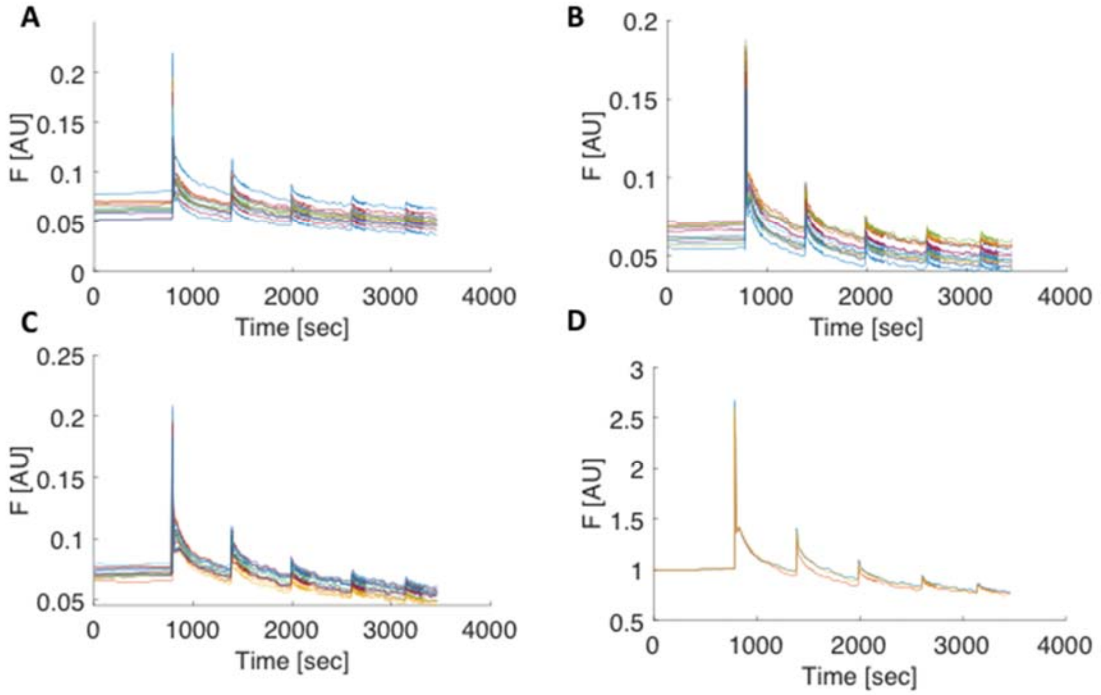

Figure S4. Individual plants for constant absolute change experiments behave similarly (Related to figure 1A). (A-C) the individual plant fluorescence in the three repeats of the absolute change experiment, each subplot is a repeat and line is a single plant. (D) The mean plant normalized fluorescence in each of the three rpeats, the results are similar in all three repeats.

Fig S5

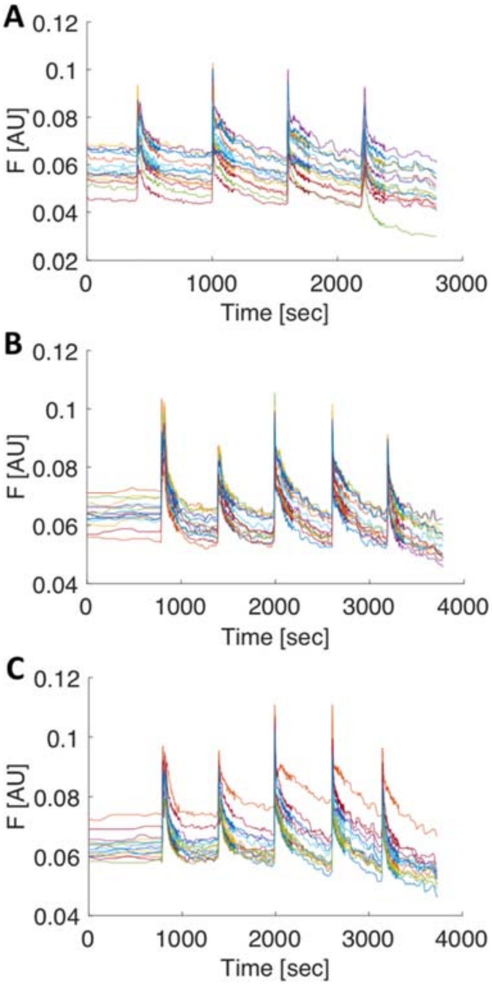

Figure S5. Individual plants for constant fold change experiments behave similarly (Related to figure 1B). The individual plant fluorescence in the three replications of the fold change experiment, each subplot is a replication and each line is a single plant. Note that the first replication started from 10uE and went up to 160uE, where replications 2 and 3 went up to 320uE.

Fig S6

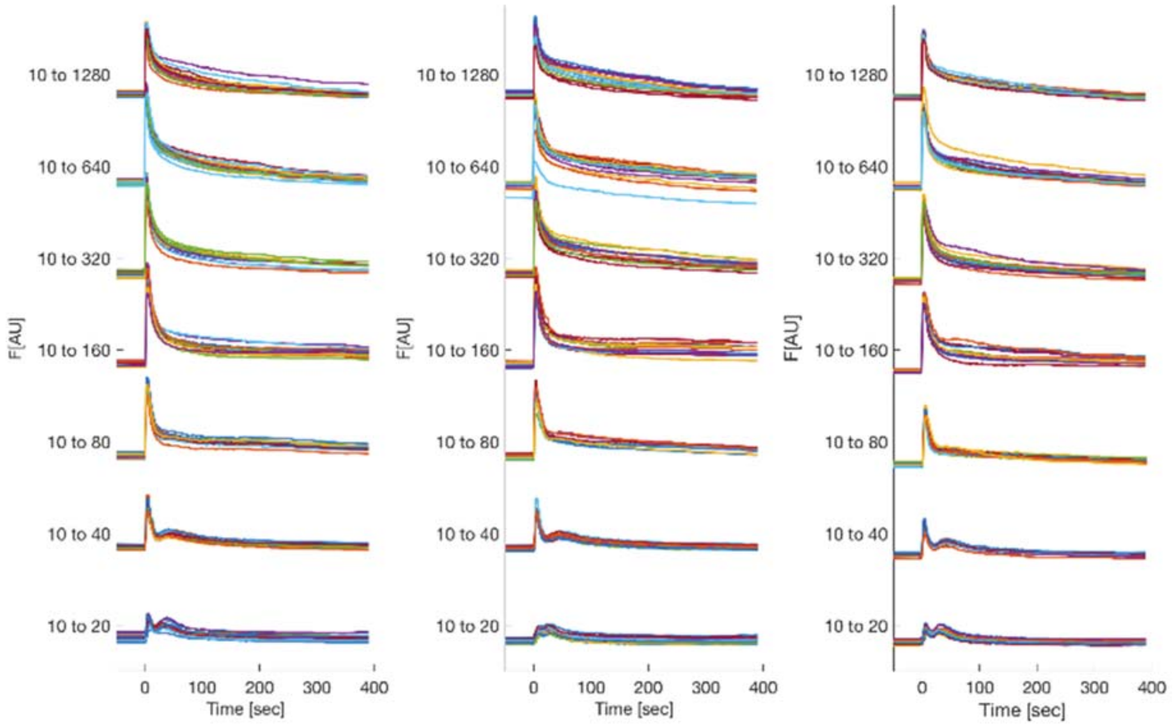

Figure S6. Individual plants on all replications of Weber-law experiments behave similarly (Related to figures 2,3). On the horizontal axis there are three repeats of each experiments and on the vertical axis there are the different experiments performed, we added different constants to the fluorescence values of the different experiments for the purpose of visualization. On the florescence axis we denoted from which experiments the data was obtained.

Fig S7

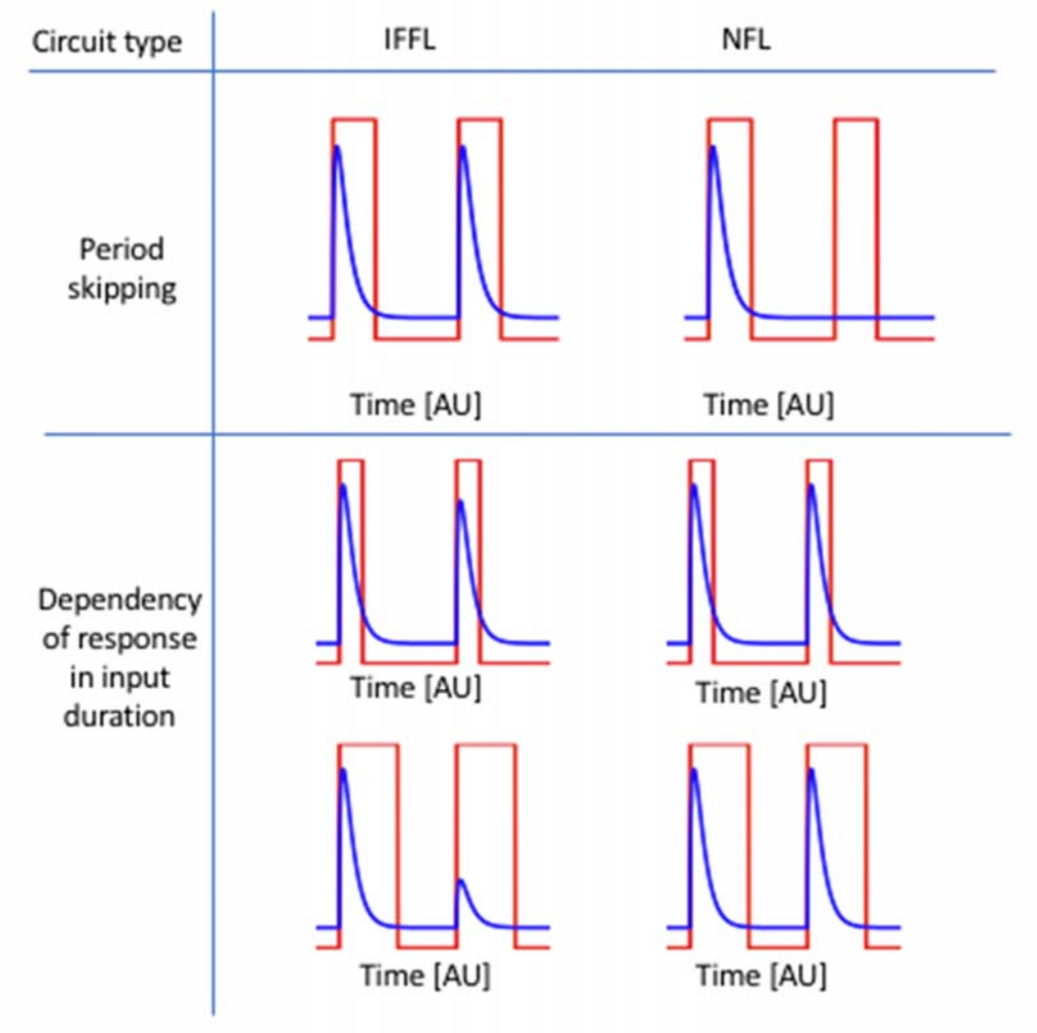

Figure S7. Exact adaptation circuits can be differentiated using input-output two-pulse experiment (Related to figure 2C). NFL can skip the response to the second pulse while IFFL cannot. For the IFFL, but not the NFL, the response to the second pulse is sometime smaller.

Fig S8

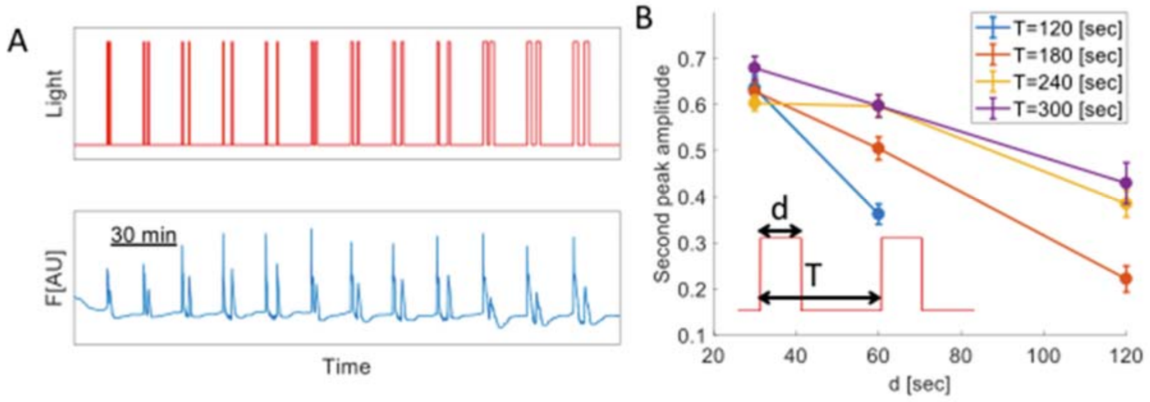

Figure S8. Two-pulse experiments indicate that the FCD mechanism is feedforward rather than feedback (Related to figure 2C). A) Plants adapted to 20uE/m2s were given pairs of 55uE/m2s light pulses. Pulses in each pair had equal duration d, with d=30,60,120s. The period of the pulse-pairs (time from start of the first pulse to the start of the second pulse) were T= 120,180,240,300s. Output fluorescence shows no second-pulse skipping. The amplitude of the second pulse was reduced relative to the first pulse. B) The amplitude of the second output pulse relative to the first pulse decreased with the input pulse duration, D. The pulse period T is indicated. Error bars are standard error of the mean over 15 plants.

Fig S9

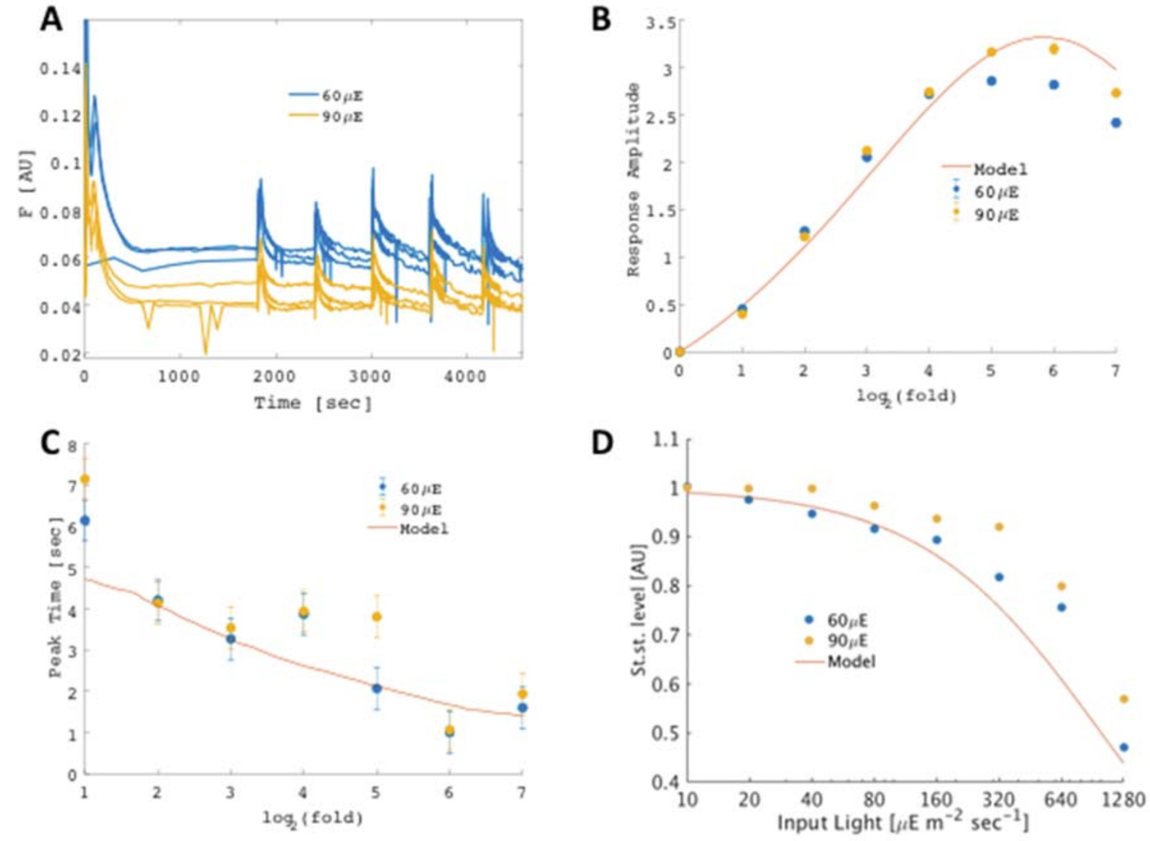

Figure S9. Plant growth condition affects mainly the fluorescence steady state and not FCD properties (Related to figure 4). We repeated the experiments in the main text with plants grown on $90\frac{\mu E}{m^2 s}$ light rather than $60\frac{\mu E}{m^2 s}$. **(A)** Three repeats of the exact adaptation experiments for plants grown on $60\frac{\mu E}{m^2 s}$ (blue) and $90\frac{\mu E}{m^2 s}$ (yellow). The steady state fluorescence for plants grown on higher light is lower in all repeats. **(B)** Response amplitude is similar in $90\frac{\mu E}{m^2 s}$ growth plant, the model is the same model as in the main text and was calibrated on $60\frac{\mu E}{m^2 s}$ plants. **(C)** The response time is also similar between the two groups of plants. **(D)** Relative change in steady state level as function of input light is also similar.

Fig S10

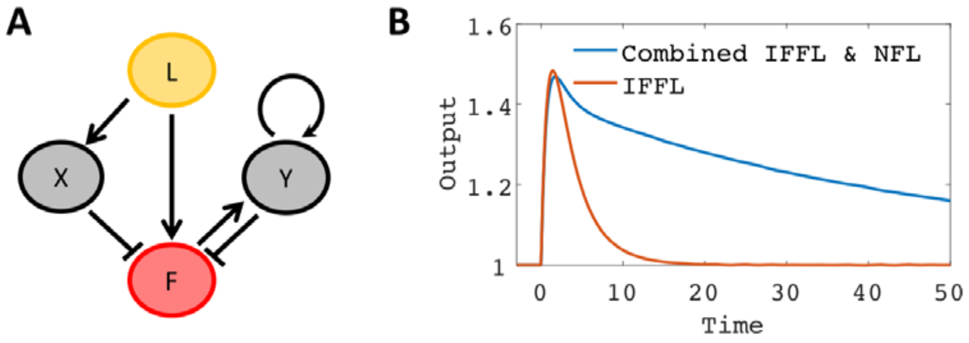

Figure S10. Combined IFFL and NFL circuit can give dynamics of two timescales. (Related to figure 2) (**A**) Diagram of combined IFFL (green arrows) and NFL circuit (yellow arrows). **(B)** Following a step input, comparison between the dynamics of a combined IFFL and NFL circuit and the dynamics of IFFL circuit, with similar response amplitude and response time. In the combined circuit we can get adaptation in two timescales, the initial adaptation is fast and is a result of the IFFL circuit, the final adaptation is slow and results from the slow timescale of the feedforward circuit. This is more similar to the experimental results.

References

Adler, M., Szekely, P., Mayo, A., and Alon, U. (2017). Optimal Regulatory Circuit Topologies for Fold-Change Detection. Cell Systems *4*, 171-+.

Rahi, S.J., Larsch, J., Pecani, K., Katsov, A.Y., Mansouri, N., Tsaneva-Atanasova, K., Sontag, E.D., and Cross, F.R. (2017). Oscillatory stimuli differentiate adapting circuit topologies. Nat Methods *14*, 1010-+.

Shoval, O., Goentoro, L., Hart, Y., Mayo, A., Sontag, E., and Alon, U. (2010). Fold-change detection and scalar symmetry of sensory input fields. Proceedings of the National Academy of Sciences of the United States of America *107*, 15995-16000.

Somvanshi, P.R., Patel, A.K., Bhartiya, S., and Venkatesh, K.V. (2015). Implementation of integral feedback control in biological systems. Wires Syst Biol Med *7*, 301-316.

Yi, T.M., Huang, Y., Simon, M.I., and Doyle, J. (2000). Robust perfect adaptation in bacterial chemotaxis through integral feedback control. Proceedings of the National Academy of Sciences of the United States of America *97*, 4649-4653.
